# Bilateral carotid body tumours: a case report from surgeon’s perspective

**DOI:** 10.1093/jscr/rjae011

**Published:** 2024-03-13

**Authors:** Kishen Raj Chandra Sakaran, Toot Tiew, Khidhir Kamil, Hisham Arshad Habeebullah Khan, Mohamad Azim Idris, Lenny Suryani Safri

**Affiliations:** Vascular Unit, Department of Surgery, Hospital Canselor Tuanku Muhriz, National University of Malaysia, Cheras 56000, Kuala Lumpur, Malaysia; Vascular Unit, Department of Surgery, Hospital Canselor Tuanku Muhriz, National University of Malaysia, Cheras 56000, Kuala Lumpur, Malaysia; Vascular Unit, Department of Surgery, Hospital Canselor Tuanku Muhriz, National University of Malaysia, Cheras 56000, Kuala Lumpur, Malaysia; Vascular Unit, Department of Surgery, Hospital Canselor Tuanku Muhriz, National University of Malaysia, Cheras 56000, Kuala Lumpur, Malaysia; Vascular Unit, Department of Surgery, Hospital Canselor Tuanku Muhriz, National University of Malaysia, Cheras 56000, Kuala Lumpur, Malaysia; Vascular Unit, Department of Surgery, Hospital Canselor Tuanku Muhriz, National University of Malaysia, Cheras 56000, Kuala Lumpur, Malaysia

**Keywords:** vascular surgery, surgery, carotid body tumour, paraganglioma

## Abstract

Carotid body tumour (CBT) is the most common paraganglioma of the head and neck and may compromise neurovascular structures such as carotid vessels, and cranial nerves. Intracranial extension from the mass is possible if left untreated. The main treatment for CBT is surgical resection albeit extremely challenging due to tumour hypervascularity and its relationship to the carotid artery. A bilateral CBT, however, is a rare occurrence. Herein, we present a case of a man who presented to us with bilateral painless and palpable neck mass. He underwent staged bilateral CBT excision and it was complicated with left hypoglossal nerve palsy, which recovered over time.

## Introduction

Extra-adrenal paragangliomas are tumour of the paraganglia which are located within the paravertebral sympathetic and parasympathetic chains. Commonly, germline mutations play a role in the development of paraganglioma such as RET, Von Hippel Lindau (VHL), Neurofibromatosis type 1 (NF1), and succinate dehydrogenase (SDH) enzyme complex subunits mutations. Some of these paragangliomas may also be hereditary in nature or comprising part of genetic syndromes such as Multiple Endocrine Neoplasms Type 2 (MEN2).

In the neck region, carotid body tumour (CBT) is the most prevalent paraganglioma and usually presents clinically as a palpable painless mass in the anterolateral aspect of the neck. The majority of these tumours are unilateral, while bilateral occurrence of CBT is a rare anomaly. In the literature, bilateral CBT was reported to occur in only 5% of all affected patients [1]. Fortunately, the majority of cases are considered to be benign, with 5 to 10% being malignant [1]. CBT occurs frequently in adults averaging 45–50 years of age and are uncommon in young age [2].

In this report, we demonstrate a case of a patient with bilateral CBT in which a two-staged CBT excision was employed.

## Case report

A gentleman with type 2 diabetes mellitus, hypertension, and a history of bilateral adrenalectomy for pheochromocytoma presented with bilateral painless neck swelling that persisted for 2 years, with no other associated symptoms. On physical examination, there was a palpable firm, fixed, round shaped mass, measuring 3 × 3 cm, with regular borders medial to the left sternocleidomastoid muscles (SCM) while over the right neck, a mass was vaguely palpable on the medial part of the right SCM, measuring 2 × 2 cm. No temperature or colour changes were present. No bruit was present over these masses.

**Figure 1:**
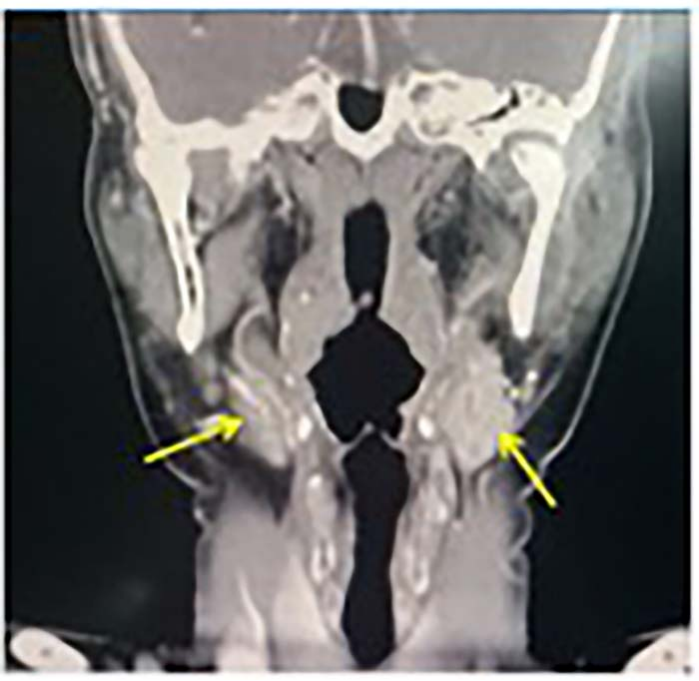
CECT neck (coronal view) view showing bilateral CBTs (arrows).

**Figure 2:**
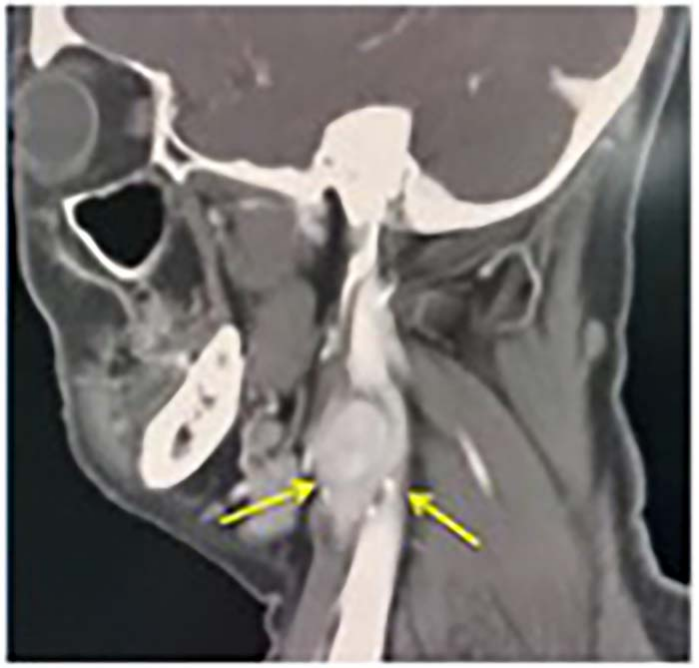
Lyre sign. Tumour in between ECA and ICA (arrows).

A contrast-enhanced computerized tomography (CECT) of the neck showed bilateral enhancing mass at the both carotid bulbs (Fig. 1), sandwiched between the external carotid artery (ECA) and the internal carotid artery (ICA) giving a positive Lyre sign. The CECT showed the left and the right mass measuring 3 × 2.5 cm and 2 × 1.2 cm, respectively (Fig. 2). No signs of thrombosis were seen. A two-staged excision was decided by the vascular surgeons and the surgical approach is described as the following.

**Figure 3:**
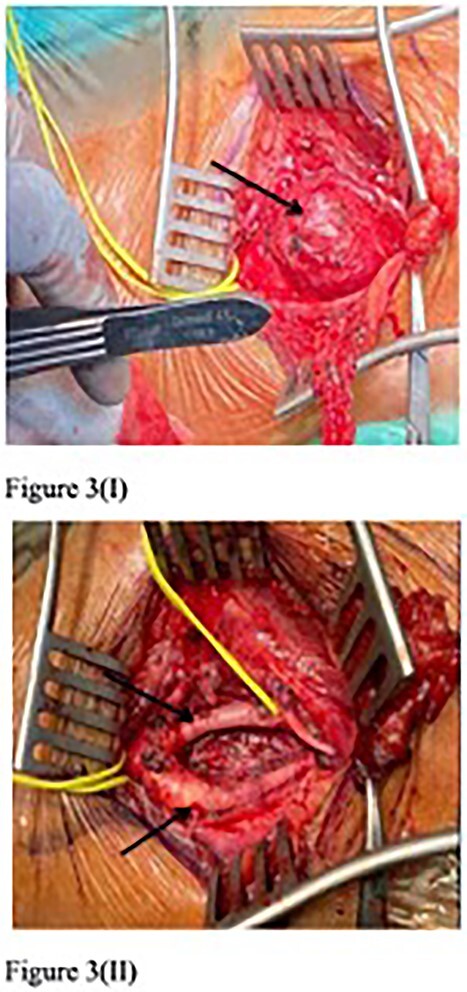
First surgery resection of left CBT (arrow) (I), with intact ECA and ICA post-operation (II).

**Figure 4:**
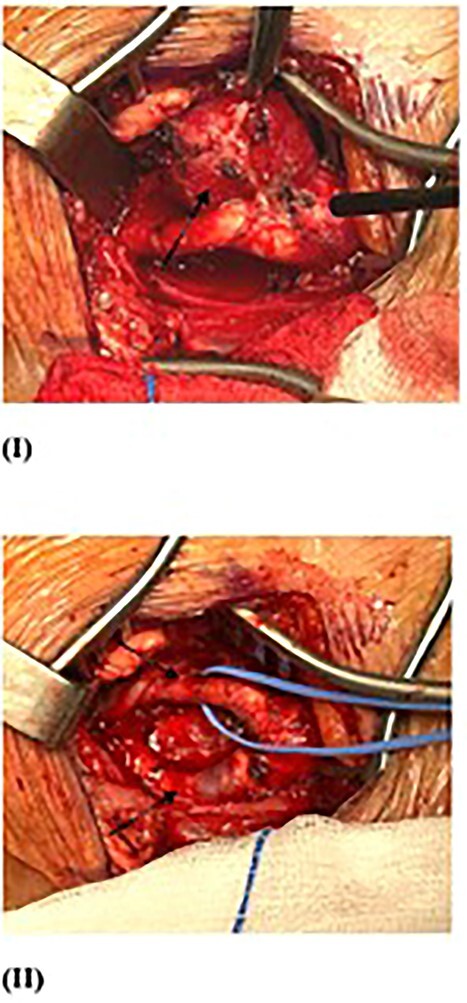
Second surgery resection of right CBT (arrow) (I), with intact ECA and ICA post-operation (II).

The first surgery involved excision of the left CBT. Initially, the bifurcation site of common carotid artery to ECA and ICA was marked over the skin. An incision was made over the left upper neck. The muscles of the neck, namely platysma, were raised and the SCM was retracted. The bifurcation of the common carotid artery then identified. Meanwhile, the hypoglossal, ansa cervicalis, and vagus nerve were preserved. Intraoperatively, the tumour adhered to the adventitia and partially to ECA and ICA, which is classified as Shamblin Type 2 (Fig. 3). The right CBT was excised 3 months later (Fig. 4).

## Discussion

This case report discussed on the approach of CBT excision in a two-staged setting in the case of bilateral occurrence. CBT is a slow growing tumour over the head and neck region with malignant potential. It occurred at any age but predominantly in adults 45–50 years [2]. Bilateral CBTs only represent ~5% of all cases and usually inherited [1]. Typically, this tumour remains asymptomatic, but as it enlarges, it can give rise to symptoms characteristic of a space-occupying lesion, including dysphagia, pain, hoarseness of voice, and stridor. Although CBT is a neuroendocrine neoplasm, it rarely produces catecholamines and typically present as a painless neck mass with slow growth.

A wide range of possible complications may arise from the surgery (Table 1). Early complications such as infection and bleeding are sometimes inevitable. Patients may have arrhythmias due to close proximity to the carotid body. Dissecting around the carotid bifurcation may give rise to thromboembolism and nerve injury. Davila et al. reported a permanent cranial nerve injury rate of 5.5% and stroke rate of 1% [3]. Amato et al. reported transient cranial nerve injury, vascular injury, permanent cranial nerve injury, stroke, and perioperative mortality at 31, 28, 17, 2.5, and 0.5%, respectively [4].

**Table 1:** Possible complications following CBT excision surgery.

**Early**	**Delayed**
Wound infection	Pseudoaneurysm
Bleeding	Chronic pain
Seroma	Hematoma
Arrhythmia	
Thromboembolism - stroke	
Injury to cranial nerves 9–11	

In the case of hyper-functional CBT, patients may present with palpitation, hypertension, headaches, arrhythmias, flushing, or even transient ischaemic stroke and stroke [5]. Although the most frequent cause of CBTs is sporadic, 30–50% of patients have hereditary predisposition [6]. Inherited head and neck paragangliomas have been associated with pathogenic variants in which different subunits of the SDH enzyme complex were encoded [6]. Symptomatic presentation, positive family history, and multiple paragangliomas have been associated with SDH gene mutation [7]. Therefore, the recognition of this mutation may facilitate long-term surveillance and identification of family members that are at risk, in which SDH mutation analysis can be offered. In our case, the patient tested positive to *SDHD* gene.

Shamblin classification is used to determine the relation of tumour towards carotid artery which has types I, II, and III. It predicts intraoperative difficulty and possible adverse neurovascular events [8]. Surgical resection remained the mainstay treatment for CBT [9]. Typically, the cure rate after complete resection of a benign CBT is 89–100% [9].

In this report, the recommended procedure to resect bilateral CBTs is a staged excision in view of the complexity of the operation itself and the increased risk of bilateral cranial nerve dysfunction from removing both tumours concurrently. Lower limb vein mapping should be considered, and vein harvesting should be anticipated if carotid artery reconstruction is needed post excision of tumour. However, if the patient is deemed unfit for surgery or having extensive multiple tumours in various body location, radiotherapy could be offered to limit disease progression. Conservative treatment can be opted for asymptomatic patients; however, it requires close follow up as the majority of the patients may be symptomatic eventually.
